# Women in chemistry: Q&A with Professor Hongjing Dou

**DOI:** 10.1038/s42004-024-01307-y

**Published:** 2024-09-30

**Keywords:** Soft materials, Bioinspired materials

## Abstract

Professor Hongjing Dou is a full Professor at the Institute of Composite Materials, School of Materials Science and Engineering at Shanghai Jiao Tong University, China, where she leads a research group focused on bionanomaterials and their biomedical applications.

Professor Hongjing Dou was born and raised in Henan, a province in central China. She earned her BSc in Chemistry and her Master’s in Polymer Materials there before moving to Shanghai in 2000 to pursue her PhD under the supervision of Professor Ming Jiang at Fudan University. After obtaining her PhD in 2003, she joined Shanghai Jiao Tong University (SJTU) as a lecturer. Her career at SJTU advanced rapidly; she was promoted to Associate Professor in 2008 and to full Professor in 2015.

**Figure:**
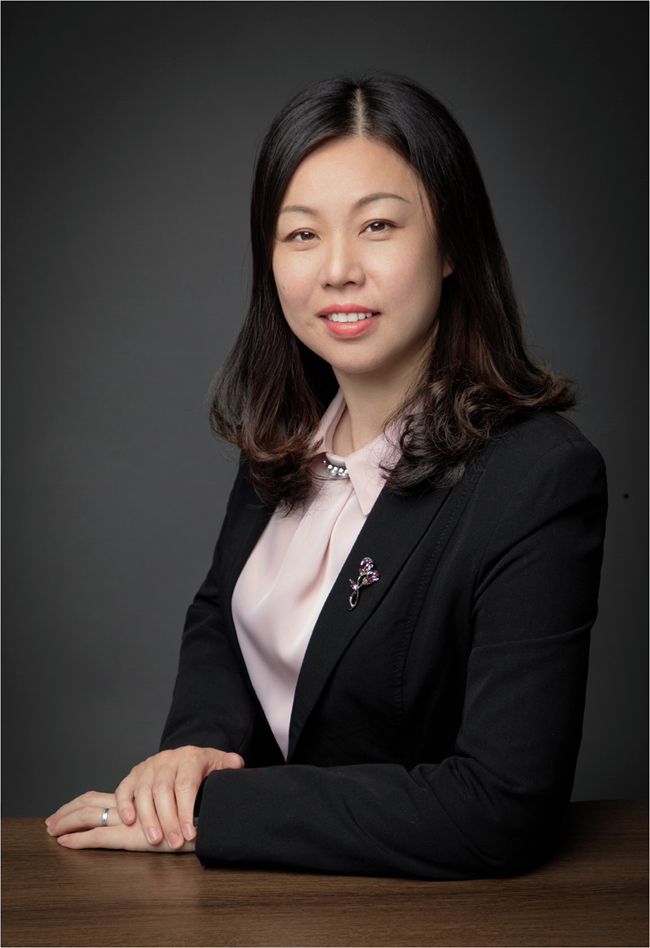
Hongjing Dou

In 2009, Professor Dou spent 14 months as a postdoctoral research fellow in Professor Guojun Liu’s group at Queen’s University in Canada. Between 2015 and 2017, she was awarded a prestigious Marie Curie International Incoming Research Fellowship, which supported her research at the University of Bristol under the guidance of the late Professor Ian Manners and Professor Stephen Mann.

In 2021, Professor Dou achieved tenure at SJTU, where she continues to contribute to the academic community. Her research focuses on the “customisation and application of bio-functional materials,” including cell-mimicking biomaterials, drug delivery systems, and cell–material interactions. She has published over 100 peer-reviewed papers and holds 20 patents. Professor Dou also serves on the editorial board of the journal *Pharmaceutics*.

Why did you choose to be a scientist?

My decision to become a scientist wasn’t shaped by a single moment or event; rather, it was the culmination of lifelong learning experiences and following my inner passion. As a child with a love for reading, I was fascinated by science and aspired to become a scientist. I fondly remember my favourite magazines being Fairy Tale King and Science Fiction World. During my university studies in chemistry at Zhengzhou University, I discovered a deep enjoyment in designing experiments and testing hypotheses, which was particularly fulfilling during my Master’s degree. At that stage, I still felt that both science and engineering were appealing paths, as they both offered the excitement of research and discovery. However, as I approached the end of my doctoral studies, I realised that my true passion lay in the freedom to explore scientific questions and delve into research driven by curiosity. Additionally, the opportunity to engage with enthusiastic students through teaching was another compelling factor in my decision to pursue a career in academia. The chance to inspire and be inspired by young minds reaffirmed my commitment to a life of scientific inquiry and education.

What scientific development are you currently most excited about?

My research focuses on creating cell-mimicking materials through the self-assembly of biomolecules and using these materials to regulate the functions of living cells via material–cell signalling. Recently, the most exciting scientific development for me has been the advancement of programmable signalling between cell-mimicking materials and living cells. This innovation opens up new possibilities for controlling the proliferation and differentiation of living cells, offering great potential for advancements in tissue engineering, regenerative medicine, and therapeutic interventions.

What direction do you think your research field should go in?

My research lies at the intersection of biomaterials and synthetic biology, bridging materials science and applied chemistry, with a strong emphasis on biomedical applications. In the future, I envision cell-mimicking materials being widely used across various fields, such as tissue and organ regeneration, disease treatment, biomass recycling, and environmental protection. My hope is that these materials will have a far-reaching impact, benefiting humanity and society as a whole. Ultimately, our goal is to see our work transition from the laboratory to practical, real-world applications, as this is where the true impact of scientific research is realised.

How would you describe your research philosophy?

Joyfully striving for excellence is the cornerstone of my research philosophy. I believe that genuine passion is the driving force behind meaningful research. I describe my approach as a “LeGo” philosophy. In Chinese, “Le” means “happiness” and “Go” represents “lofty goals,” which perfectly align with the idea of joyfully striving for excellence. By maintaining a sense of joy and aiming for high standards, I believe we can push the boundaries of knowledge and achieve significant breakthroughs.

What impact has your gender had on your career as a scientist?

Although I am a woman, my parents always encouraged me to pursue my career and life goals without limitations, so gender has never been a confining factor in my thinking. On the contrary, I believe that qualities often associated with women—such as meticulousness, patience, calmness, nurturing, and strong communication skills—have positively influenced my career. These attributes have helped me to effectively support and understand students and colleagues of different ages and personalities, and to appreciate the diverse perspectives and ideas of my collaborators. This openness and adaptability have been invaluable in my scientific journey.

What action(s) do you feel employers in chemical research should take to make a difference for women scientists?

Many women scientists, in addition to their research responsibilities, often take on greater family responsibilities after marriage and having children. This is a significant reason why fewer women are represented among top scientists. To better support women in science, employers should consider implementing measures such as extended paid maternity leave, flexible part-time working arrangements, and ensuring a fair proportion of women recipients in research funding applications. Personally, I have received tremendous support and opportunities from my institution, Shanghai Jiao Tong University, which has created a nurturing environment for women in science. The establishment of various networking organisations within the university has been particularly effective in supporting and promoting women scientists.

How can publishers, editors, funders, and conference organizers better support women scientists?

Publishers can encourage more women scientists to join editorial boards and allocate dedicated space for publishing comments, viewpoints, and scientific papers authored by women. Funders can offer greater support by creating special research grants for women scientists and ensuring a higher proportion of women beneficiaries in funding opportunities. Conference organisers can support women scientists by establishing dedicated women’s forums and actively involving women as organisers and speakers at conferences.

What steps do you feel are needed to retain more women in chemistry, for example from PhD level to Professorship?

To retain more women in chemistry from the PhD level to professorship, it is essential to create more opportunities that encourage them to aim for top scientific positions. The power of role models cannot be overstated; having visible female leaders in academia can inspire young women to pursue research careers after their PhDs. Support for women scientists should be prioritised, especially at the early stages of their careers, such as during doctoral and postdoctoral training. This could involve the creation of targeted postdoctoral fellowships and research grants specifically for young female scientists. These initiatives would provide crucial financial backing and recognition, helping to build their confidence and encouraging them to pursue academic advancement.

Mentorship programmes are also vital, offering guidance, advice, and support networks that can help navigate the challenges often faced in academia. Additionally, implementing policies that promote work-life balance, such as flexible working hours and childcare support, would make it easier for women to balance professional ambitions with personal responsibilities. By fostering a supportive environment and providing tailored resources, we can encourage more women to continue their careers in chemistry, ultimately contributing to a more diverse and innovative scientific community.

*This interview was conducted by the editors of Communications Chemistry*.

